# Onset of meiosis in the chicken embryo; evidence of a role for retinoic acid

**DOI:** 10.1186/1471-213X-8-85

**Published:** 2008-09-17

**Authors:** Craig A Smith, Kelly N Roeszler, Josephine Bowles, Peter Koopman, Andrew H Sinclair

**Affiliations:** 1 Murdoch Childrens Research Institute and University of Melbourne Department of Paediatrics, Royal Children's Hospital, Melbourne, 3052, Australia; 2 Institute for Molecular Bioscience, University of Queensland, 4072, Brisbane, Australia

## Abstract

**Background:**

Meiosis in higher vertebrates shows a dramatic sexual dimorphism: germ cells enter meiosis and arrest at prophase I during embryogenesis in females, whereas in males they enter mitotic arrest during embryogenesis and enter meiosis only after birth. Here we report the molecular analysis of meiosis onset in the chicken model and provide evidence for conserved regulation by retinoic acid.

**Results:**

Meiosis in the chicken embryo is initiated late in embryogenesis (day 15.5), relative to gonadal sex differentiation (from day 6). Meiotic germ cells are first detectable only in female gonads from day 15.5, correlating with the expression of the meiosis marker, SCP3. Gonads isolated from day 10.5 female embryos and grown in serum-free medium could still initiate meiosis at day 16.5, suggesting that this process is controlled by an endogenous clock in the germ cells themselves, and/or that germ cells are already committed to meiosis at the time of explantation. Early commitment is supported by the analysis of chicken *STRA8*, a pre-meiotic marker shown to be essential for meiosis in mouse. Chicken *STRA8 *is expressed female-specifically from embryonic day 12.5, preceding morphological evidence of meiosis at day 15.5. Previous studies have shown that, in the mouse embryo, female-specific induction of *STRA8 *and meiosis are triggered by retinoic acid. A comprehensive analysis of genes regulating retinoic acid metabolism in chicken embryos reveals dynamic expression in the gonads. In particular, the retinoic acid-synthesising enzyme, *RALDH2*, is expressed in the left ovarian cortex at the time of *STRA8 *up-regulation, prior to meiosis.

**Conclusion:**

This study presents the first molecular analysis of meiosis onset in an avian embryo. Although aspects of avian meiosis differ from that of mammals, a role for retinoic acid may be conserved.

## Background

One of the most fundamental developmental events in vertebrate embryos is the sexually dimorphic regulation of germ cell fate. In male mammals, the germ cells become incorporated into developing seminiferous cords of the testis during embryogenesis, where they enter mitotic arrest as prospermatogonia [1]. This quiescent state is thought to be induced by a signal from the adjacent Sertoli cells of the testis cords [2]. In contrast, germ cells in the female gonad enter the early stages of meiosis during embryonic life, arresting at prophase I as oocytes. Early studies showed that germ cells of either sex can enter meiosis if they migrate to ectopic sites such as the adrenal gland [3] or if isolated and cultured *in vitro *[4]. A long held view based on these observations was that germ cells enter meiosis cell-autonomously if they do not become enclosed in testis cords, as in males. An alternative possibility is that meiosis is induced, and that the inductive signal in developing ovaries is also present in non-gonadal sites and in culture conditions *in vitro*. Recent studies have now shown that meiosis is indeed induced in the mouse embryo, and that the inductive signal is retinoic acid [5,6].

A major retinoic acid synthesising enzyme in the mammalian embryo is Raldh2 (Retinaldehyde dehydrogenase, type 2; also called Aldh1a2) [7]. Sites of *Raldh2 *mRNA expression in the embryo precisely reflect sites of retinoic acid production. In the embryonic mouse urogenital system, *Raldh2 *mRNA is expressed in the mesonephric kidneys rather than the gonads themselves, and retinoic acid is then thought to diffuse into the neighbouring gonad [5]. Sexual dimorphism in retinoic acid availability, however, is achieved through the differential expression of *Cyp26b1*, which encodes a major retinoic acid-degrading enzyme. In developing mouse gonads, *Cyp26b1 *is male up-regulated in the gonads from embryonic day 12.5, just prior to germ cell mitotic arrest [5,8]. In contrast, *Cyp26b1 *expression disappears in female gonads at the time of meiosis [5]. This results in the accumulation of retinoic acid in fetal ovaries and the induction of the pre-meiotic protein, Stra8, which is essential for meiosis [9,10]. Male mouse gonads cultured in the presence of retinoic acid show up-regulation of *Stra8 *and expression of meiosis markers such as *Synaptonemal complex 3 *(*Scp3*). Furthermore, meiosis markers are activated in male mouse gonads cultured with CYP enzyme inhibitors such as ketoconazole (retinoic acid is therefore not degraded) or in gonads derived from *Cyp26b1 *null mutant mice [5,6,11]. Conversely, treatment of cultured gonads with a retinoic acid antagonist blocks or retards germ cell meiosis in females [5,6]. Altogether, these data indicate that retinoic acid induces meiosis in female mouse gonads, and that meiosis is prevented in male embryos by the degrading enzyme, Cyp26b1.

In this study, we describe the onset of meiosis at the molecular level in an avian model, the chicken embryo. Chicken germ cells originate at an extra-gonadal site and migrate into the gonads via the bloodstream. They settle in the gonads by day 6 of the 21 day incubation period, with more cells populating the left versus the right gonad (in both sexes) [12]. Male germ cells do not proliferate significantly from the time of testicular organization (day 6 onwards), while female germ cells proliferate considerably, from at least as early as day 9 [13,14]. In the female, germ cell fate differs between the left and right gonads. In the left gonad, a thickened outer cortex of somatic cells develops, and nests of synchronously proliferating germ cells become apparent in the left cortex from day 10. It has been estimated that the total population of germ cells in the left ovarian cortex increases about twenty-five fold between day 9 and day 17 of incubation [14]. In contrast, the right gonad fails to proliferate a cortex. Germ cells in the right gonad proliferate somewhat in the underlying medulla, but they later undergo apoptosis [15].

We find that meiosis only occurs in those germ cells located in the left ovarian cortex, from day 15.5 of incubation. The premeiotic marker, *STRA8*, is female up-regulated 2–3 days prior to this time (day 12.5). In addition, we provide evidence that meiosis in birds may also depend upon retinoic acid signalling, as in mouse. In the chicken embryo, *RALDH2 *is expressed in the gonads themselves, rather than the mesonephric kidneys (as is the case in the mouse). In females, *RALDH2 *expression is specifically localised in the left gonadal cortex at the time of *STRA8 *expression, and the cortex is the site of meiosis. *CYP26B1 *(which degrades retinoic acid) is expressed in both sexes, but not in the outer cortical layer of developing ovaries, where meiosis occurs. In the right female gonad (which lacks a cortex) *RALDH2 *and *CYP26B1 *are both expressed in the gonadal medulla, potentially leading to RA synthesis and degradation. These observations suggest a conserved role for retinoic acid in vertebrate meiosis.

## Methods

### Embryos and histology

Fertile chicken eggs (White Leghorn × Australop cross) were incubated at 37.8°C under humid conditions. Embryos were staged according to the morphological criteria of Hamburger and Hamilton [16], and sexed by PCR, using W (female)-specific *Xho1 *primers and 18S rRNA primers as internal controls [17]. For histology, urogenital systems were dissected from embryos and fixed in Bouin's solution, followed by dehydration in graded ethanols. Tissues were then infiltrated and embedded in paraffin. Six micron sections were mounted onto gelatin-coated slides and stained with haematoxylin and eosin using standard methods. Germ cells were recognised by their relatively large size and pale cytoplasm. The nuclei of meiotic cells showed condensed, thread-like chromatin.

### Whole mount *in situ *hybridisation

Whole mount *in situ *hybridisation was used to examine the expression of genes encoding meiosis markers and enzymes involved in retinoic acid production and catabolism. In the mouse, *Stra8 *(Stimulated by retinoic acid, number 8) is a premeiotic marker, and the germ cells of *Stra8 *null mutant mice fail to enter meiosis [9]. An 816 base pair fragment of the predicted chicken *STRA8 *sequence (XM_416179) was amplified by PCR, using mixed sex embryonic gonadal cDNA. The *cSTRA8 *primers were *cSTRA8*-For: 5'-TATCCAGGAGCTGGAGCAAACC-3' and *cSTRA8*-Rev: 5'-TCAAAGGTCTCCGTGCACCG-3'. A Chicken 750 bp *Synaptonemal Complex protein 3 *(*SCP3*) fragment was PCR amplified from day 16.5 female gonadal cDNA, using the following specific primers: c*SCP3*-For: 5'-CTCAGCAGCAGATCTTTGCAGC-3' and c*SCP3*-Rev: 5'-GGTACAAGTTGTTTCTCCATTGAGCC-3'. cDNA clones of chicken *RALDH1*, *RALDH2*, *RALDH3*, *CYP26A1*, *CYP26B1*, *CYP26C1 *were gifts from Malcolm Maden, King's College, London [18]. The identity of clones was confirmed by sequencing.

Whole mount *in situ *hybridisation was carried out as described previously [19], with some modifications. Briefly, whole urogenital systems were dissected in chilled DEPC-treated PBS, fixed overnight at 4°C in 4% paraformaldehyde (PFA)/PBS and dehydrated in graded methanols on ice. Following rehydration, tissues were incubated in PBS containing 0.1% Triton X-100 (PBTX), and then permeabilised with a high concentration of proteinase K in PBTX (50 μg/mL) for 45 to 120 minutes at room temperature. Permeabilisation times varied according to the age of the tissues: embryonic day 8.5 – 12.5 urogenital systems were permeabilised for 45–75 minutes, and older tissues, (day 14.5–16.5) were permeabilised for up to 120 minutes. Antisense and sense digoxygenin-labelled riboprobes were synthesised from PCR generated cDNA templates, precipitated and added to tissues equilibrated in prehybridisation buffer. Hybridisation was carried out overnight at 65°C, following by washing in 2× SSC/0.1% CHAPS and 0.2 × SSC/0.1% CHAPS (30 minutes each at 65°C). Tissues were then pre-blocked in TBTX (50 mM Tris-HCl, pH7.5, 150 mM NaCl, 0.1% Triton X-100) containing 10% sheep serum and 2% BSA (TBTX/NSS/BSA), then incubated overnight in TBTX/NSS/BSA containing alkaline phosphatase-congujated anti-digoxygenin antibody (1:200). Following extensive washing in TBTX/0.1%BSA, tissues were incubated in NTMT buffer containing chromogen (BCIP/NBT). Stained tissues were photographed under a Leica MZ8 stereomicroscope. To visualise staining in sections, some tissues were then left in chromogen solution over two days at room temperature, cryoprotected in 30% sucrose in PBS (overnight at 4°C), snap frozen in OCT embedding compound, cut on a cryostat at 14 μm and photographed. When *in situ *hybridisation was used to compare expression in male and female samples, all tissues were treated in parallel including being stained for the same length of time.

### Isolation of RNA and real time PCR (qRT-PCR)

Real time PCR was used to assess the expression of chicken *STRA8*, *SCP3*, *RALDH2 *and *CYP26B1*. Pools of paired gonads were dissected from staged embryos over embryonic days 6.5 to 16.5 and total RNA was extracted using Trizol^® ^reagent (Invitrogen). Ten micrograms of RNA was firstly treated with DNase1 using DNA-Free (Ambion) and 1 μg was then reversed transcribed using random hexamers together with oligo dT as primers. All samples included RT minus reactions to control for contaminating genomic DNA. For real time PCR, 1 μl (50 ng cDNA) was used in each reaction. All PCRs were carried out according to standard protocols. Real time PCR was performed using the Universal Probe Library (UPL) system and Faststart Taq Probe Master mix with ROX (Roche). This method involves the generation of standard curves for each primer pair, to test and account for variations in primer efficiency. Fifty nanograms of cDNA was subject to amplification using an ABI 7900 HT Real Time PCR machine. Individual samples were analysed in triplicate and experiments performed twice. All samples were normalised against *HPRT *using the comparative C_T _method (ΔΔC_T_). Results were plotted as percentage of maximum expression+/- SEM. Standard curves were constructed for each gene and high PCR efficiency (> 0.90%) was confirmed. Cycle parameters and primers for real time PCR primer/UPL probe set combinations used are included in the supplementary information.

### Immunofluorescence

Immunofluorescence was performed as described previously [20]. Most tissues were fixed in 1% or 4% paraformaldehyde in PBS at room temperature for 10–20 minutes, cryoprotected in 30% sucrose/PBS overnight at 4°C, immersed in OCT embedding compound and snap frozen. Ten micron frozen sections were permeabilised in 1% Triton X-100/PBS for 10 minutes. In the case of SYN/COR staining, unfixed frozen sections were postfixed with -20°C methanol for 10 minutes. All sections were then blocked for 1 hour in 2% BSA/PBS. Primary antibodies were diluted in 1% BSA/PBS and added overnight at 4°C. Rabbit anti- SYN/COR antibody, a marker of meiosis, was obtained from Barbara Spyropoulos and Peter Moens, (York University, Toronto) and was used at 1:500. Rabbit anti-chicken RALDH2 antibody was obtained from Dr. Shan Sockanathan (Johns Hopkins University Medical School, Baltimore) and used at 1:500. Rabbit anit-SPC3 was obtained from Novus Biologicals (1:500). Alexa Fluor secondary antibodies were obtained from Molecular Probes Inc. and used at 1:500 – 1:1500. Sections were routinely counterstained with the nuclear stain, DAPI (300 nM). Negative control sections were incubated with pre-immune serum instead of primary antibody, or with secondary antibody alone. In all such cases, no specific staining was observed.

### Organ culture

To study meiosis *in vitro*, individual gonads (left ovaries or testes), with or without the mesonephros, were explanted at embryonic day 10.5 (stage 36). Tissues were placed onto 5 μm isopore filters (Millipore), floating on 500 μL of culture medium. The culture medium was either serum-supplemented (DMEM + 10% foetal calf serum + 2% chick serum) or serum deprived (DMEM + insulin transferrin, sodium selenium supplement (ITS)). Ampicillin (50 μg/mL) was added to all cultures, and fresh medium was added daily. After 6 days in culture at 37°C/5% CO_2_, tissues were fixed in 4% paraformaldehyde in PBS (30 minutes at room temperature), cryoprotected overnight in 30% sucrose/PBS, and 10 μm frozen sections cut for immunofluorescence as described above.

## Results

### Histological and molecular characterisation of meiosis onset in the chicken embryo

**Figure 1:**
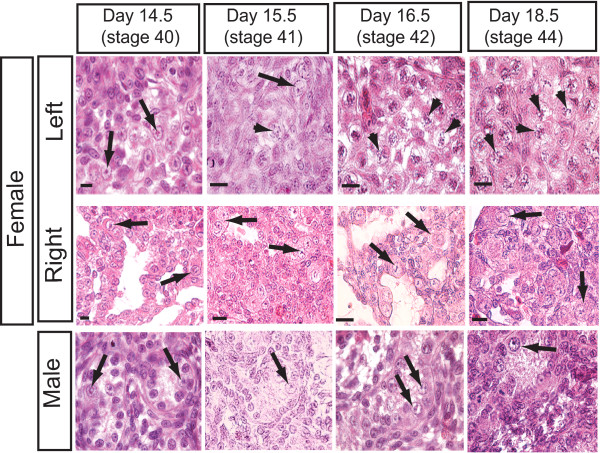
**Timing and morphology of meiosis in embryonic chicken gonads, assessed by haematoxylin and eosin staining. Arrows indicate non meiotic germ cells; arrowheads indicate meiotic germ cells.** At day 14.5 (stage 40) non-meiotic germ cells are present in the gonads of both sexes, recognised by their large size and pale cytoplasm. Meiotic figures are first seen in some germ cells of left female gonads at day 15.5 (arrowheads). The chromatin becomes condensed and thread-like. By day 18.5, most germ cells are meiotic in the left female gonad. In the right female gonad and in both male gonads, only non-meiotic germ cells are present throughout development. Scale bar = 20 μm.

The onset of meiosis in the chicken embryo was assessed histologically by haematoxylin and eosin (H & E) staining of serial sections through the developing gonads. Embryonic chicken gonads of both sexes initially comprise two regions, an inner layer of somatic cell cords (the medulla) and an outer epithelial layer, the cortex. The germ cells migrate into the gonads prior to gonadal sex differentiation. They are scattered throughout the gonad, but are more prevalent in the outer cortical layer. During male development, the inner medulla gives rise to seminiferous cords in both gonads. In female embryos, the outer cortical layer of the left gonad thickens and germ cells accumulate there, while the right gonad fails to proliferate a cortex and germ cells remain scattered in the medulla [21]. Gonadal sex differentiation begins at embryonic day 6.0 (stage 29) [22]. However, in the study described here, the first signs of meiosis were not detected until much later in development. Meiosis was only seen in female gonads, with morphological evidence of meiosis first detectable in the outer layer (cortex) of developing left ovaries at day 15.5 (stage 41). Germ cells were recognised by their large size (≥ 20 μm) and pale cytoplasm relative to somatic cells. Meiotic germ cells were distinguished by the presence of condensed thread-like chromatin, as shown in Fig. 1. At day 16.5 (stage 42), meiotic germ cells were more numerous and, by day 18.5 (stage 44) they were abundant in the left gonad. In contrast, germ cells in the right gonad did not exhibit meiotic figures (Fig. 1). No meiotic germ cells were detected in the developing testes of male embryos, as assessed by H&E staining (Fig. 1).

**Figure 2:**
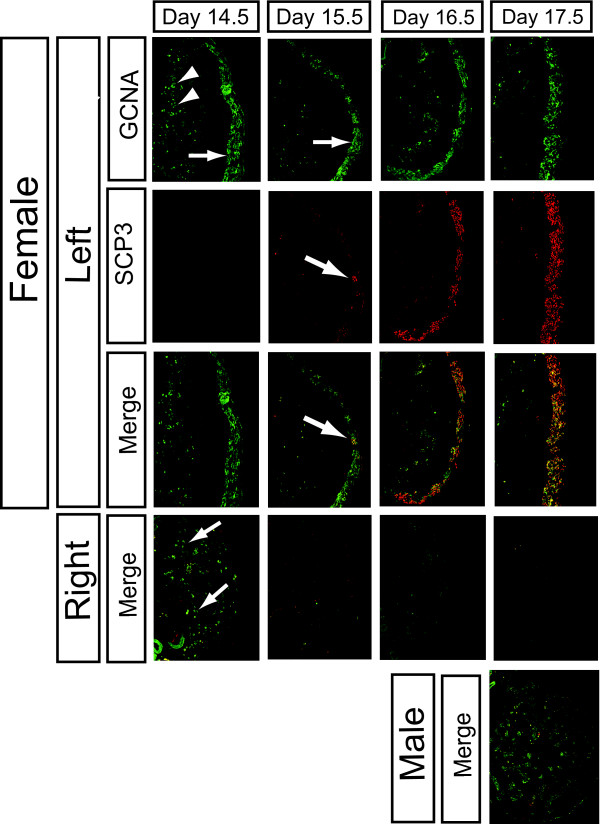
**Expression of Synatonemal Complex 3 protein (SCP3) in embryonic chicken gonads, assessed by immunofluorescence.** Germ cells are recognised by their immunoreactivity to GCNA antibody (green). In the left female gonad, most germ cells are concentrated in the outer cortex (arrows), but some are present in the inner part of the medulla (arrowheads). Some SCP3 positive cells (red) are first detectable in the cortex of the left female gonad at day 15.5 (arrow), increasing in number up to day 17.5. No SCP3 positive cells are detectable in the left medulla, the right gonad, or in male gonads. In the right female gonad, GCNA staining (green; arrows) is gradually lost after day 14.5.

**Figure 3:**
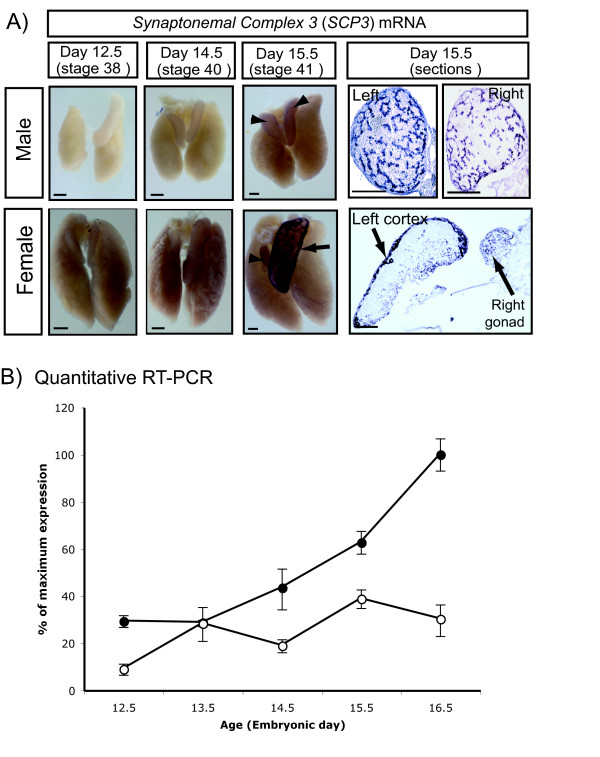
**Expression of *SCP3 *mRNA in embryonic chicken gonads**. A) Whole mount *in situ *hybridisation. Strong *SCP3 *mRNA expression is seen in the left and right female gonads from day 15.5 (arrow and arrowhead). A lower level of expression is detectable in male gonads at the same time (arrowheads). Over-stained and sectioned whole mounts show bilateral expression in the seminiferous cords of developing testes. In females, mRNA expression is localised to the cortex of the left gonad, and in scattered cells of the left and right medulla. Scale bar on whole mounts = 1 mm, scale bar on sections = 100 μm. B) Quantitative real time PCR (qRT-PCR) for *SCP3 *mRNA expression. A low level of expression is detectable in male gonads throughout development (open circles), while expression increases in female gonads during the time of meiosis (closed circles).

The sex-specificity of meiosis was confirmed by immunofluorescent detection of synaptonemal complex 3 (SCP3), a meiosis structural protein that was expressed in female but not male embryonic gonads (Fig. 2). In females, germ cells were detected in the cortex of the left gonad and scattered in the medulla of the right gonad at day 14.5, as assessed by immunoreactivity to GCNA, a cell surface germ cell marker (Fig. 2). A small number of GCNA positive cells in the developing cortex became SCP3 positive at day15.5, and, by day 16.5, SCP3 protein expression was widespread (Fig. 2). In contrast, germ cells scattered in the right female gonad initially expressed GCNA but not SCP3 protein, indicating that germ cells of the right gonad do not enter meiosis. GCNA expression was gradually lost in the right gonad. Male gonads were positive for GCNA but not SCP3 protein (Fig. 2). The SCP3 staining was corroborated by independent staining for SYN/COR, another meiosis marker (not shown). *SCP3 *expression was also examined at the transcriptional level by whole mount *in situ hybridisation *and quantitative (real time) RT-PCR (Fig. 3). *SCP3 *transcripts were first detected by in situ hydridisation at day 15.5, consistent with the histology, indicating meiosis onset at this time. However, expression was observed in both sexes, but was stronger in females (Fig. 3). Sections of over-stained whole mounts revealed *SCP3 *mRNA in the outer cortical region of the left ovary, the site of meiosis, in agreement with SCP3 protein immunofluorescence. In the right female gonad, *SCP3 *mRNA expression was distributed throughout. In sectioned males whole mounts, *SCP3 *mRNA was detectable in the seminiferous cords of both gonads from day 15.5 (Fig. 3). Expression of *SCP3 *mRNA in both sexes was confirmed by qRT-PCR. Expression was low in males, but became elevated in females during meiosis. (Fig. 3B). However, in males, no *SCP3 *mRNA was protein was detected (refer to Fig. 2). Taken together with the immunofluorescence results, these data indicate that *SCP3 *mRNA is expressed in both sexes, but more highly in females, while the transcript is only translated in meiotic cells – that is, in germ cells of the left ovarian cortex.

### Chicken STRA8 expression

**Figure 4:**
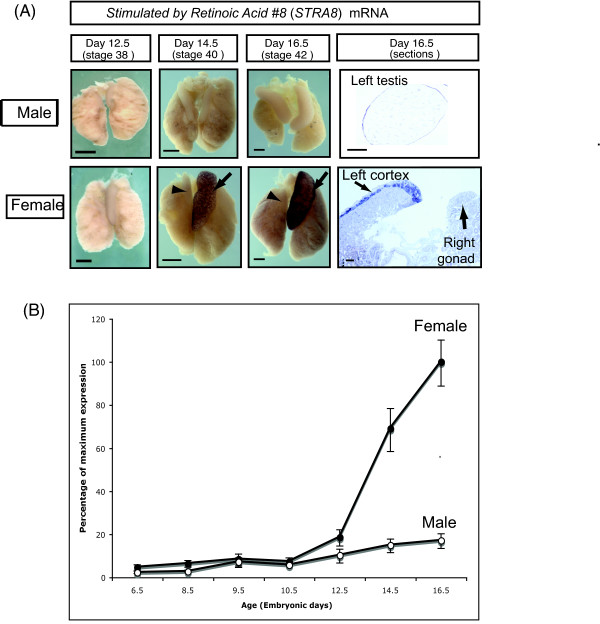
***STRA8 *mRNA expression in embryonic chicken gonads, as assessed by whole mount *in situ *hybridisation and quantitative real time PCR (qRT-PCR)**. (A) In females, expression is specific to cortex of the left gonad, and is first detectable by whole mount *in situ *hybridisation at day 14.5. No *STRA8 *mRNA expression is detectable in male gonads. Scale bar in whole mounts = 1 mm, scale bar in tissue sections = 100 μm. (B) *STRA8 *qRT-PCR. *STRA8 *is up-regulated female-specifically from day 12.5. A low level of expression is detectable in male gonads throughout development. Mean +/- SEM.

A critical gene involved in the induction of meiosis in mammals is *Stra8 *(*St*imulated by *r*etinoic *a*cid, #*8*) [9]. *STRA8 *is considered to be a pre-meiotic germ cell marker. Analysis of avian *STRA8 *has not hitherto been reported. Cloning and analysis of chicken *STRA8 *revealed female specific expression in the developing left ovary that preceded the onset of meiosis at the histological level (Fig. 4). In female embryos, *STRA8 *mRNA expression was first detectable in the left gonad by whole mount *in situ *hybridisation at day 14.5 (stage 40), one day prior to the onset of meiosis at day 15.5 (stage 41). Using whole mount *in situ *hybridisation, No *STRA8 *mRNA was detected in male gonads at any stage examined (day 6.5 – day 16.5). In tissue sections of over-stained whole mounts, *STRA8 *mRNA was localised in the left ovarian cortex (Fig. 4), the site of meiosis as demonstrated by histology and SCP3 staining. No *STRA8 *expression was detectable in the right female gonad, consistent with the lack of meiosis there (Fig. 4). Quantitative RT-PCR (qRT-PCR) confirmed elevated *STRA8 *mRNA expression in female gonads. (Both left and right gonads were pooled and assayed together.) By qRT-PCR, female up-regulation of STRA8 was first detectable at day 12.5 (stage 38) (Fig. 4B). These results indicate that *STRA8 *is a premeiotic marker in the chicken, as in mouse.

### Meiosis *in vitro*

**Figure 5:**
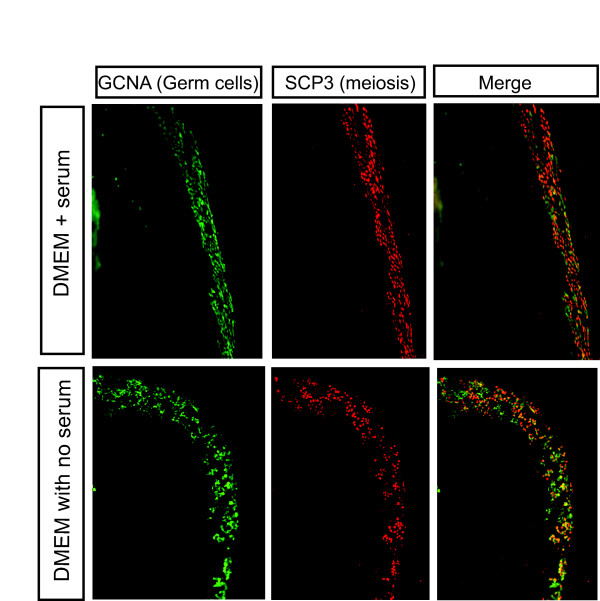
**Meiosis in female chicken embryonic gonads *in vitro*.** Day 10.5 embryonic gonads were explanted and grown in the absence or presence of serum (DMEM+ 10% FCS, 2% chick serum). After six days in culture, meiosis occurred normally in the developing cortex, as assessed by SCP3 immunofluorescence (red) in GCNA+ germ cells (green).

To determine whether germ cells could initiate meiosis *in vitro*, urogenital systems (gonads+mesonephric kidneys) were excised from embryos and cultured *in vitro *for up to 6 days. Gonads were then assayed for expression of the meiosis marker protein, SCP3. Gonads explanted on day 10.5 or 12.5 and cultured to E16.5 in the presence or absence of serum could initiate meiosis *in vitro *(Fig. 5). GCNA^+ ^germ cells in female gonads were also largely SCP3 positive. Germ cells in male gonads were SCP3 negative, as *in vivo *(not shown). Induction of meiosis in basal, serum-free medium, and hence in the absence of any exogenous signaling or growth factors, indicates that female germ cells may enter meiosis cell autonomously, or that the cells are committed to the female pathway by the time of explantation, that is, day 10.5. The latter implies that a signal for meiosis is activated at or before E10.5. Attempts were made to culture gonads from younger embryos (day 6.5 or day 9.5) but extensive germ cell loss occurred.

### Dynamic regulation of retinoic acid metabolism in the embryonic gonads

**Figure 6:**
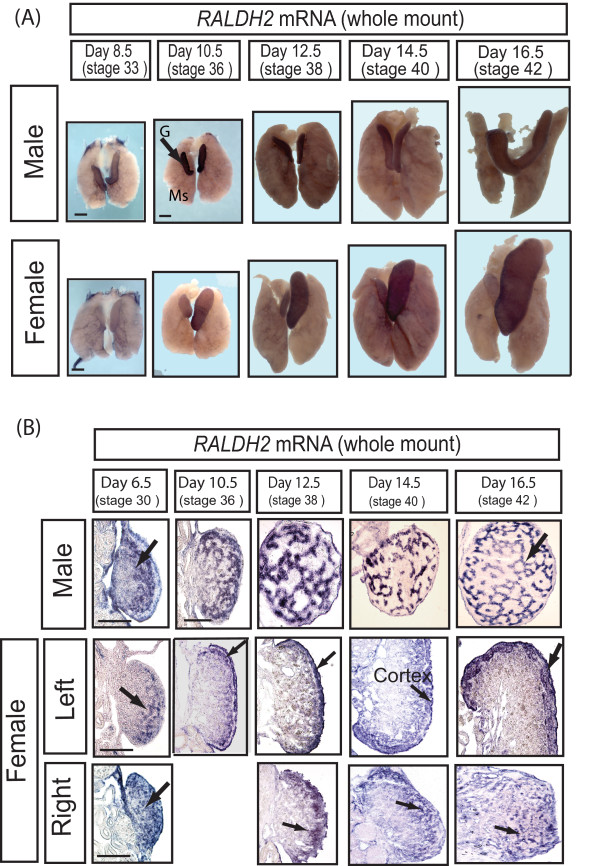
**Expression of *RALDH2 *mRNA in embryonic chicken gonads, as assessed by whole mount *in situ *hybridisation**. (A) In both sexes, expression is detectable in both gonads throughout development (e.g, G). There is no significant expression in the mesonephric kidneys (eg. Ms). (B) In males, *RALDH2 *expression localises to the seminiferous cords. In females, expression becomes localised in the proliferating cortex of the left ovary (arrows) and in the medulla of the right gonad (arrows). Scale bar in whole mounts = 1 mm, scale bar in tissue sections = 100 μm.

We next examined the expression of enzymes responsible for retinoic acid production in embryonic chicken gonads to test for a possible conserved role in regulating meiosis. Retinoic acid synthesis in vertebrate embryos is tightly regulated by the tissue specific expression of three synthetic enzymes, RALDH1, RALDH2 and RALDH3. The expression of these enzymes is coordinated with specific expression of three retinoic acid-degrading enzymes, CYP26A1, CYP26B1 and CYP26C1. RALDH2 is the major enzyme catalysing retinoic acid synthesis in the avian embryo, and sites of *RALDH2 *gene expression correlate with retinoic acid production and release [23]. We found that, in embryonic chicken urogenital systems, *RALDH2 *mRNA was robustly expressed in the gonads of both sexes at all stages examined, including the period of meiosis in females (Fig. 6A). Little if any *RALDH2 *expression was detected in the adjoining mesonephric kidneys. Gonadal *RALDH2 *mRNA was detectable by whole mount *in situ *hybridisation as early as day 6.5 (onset of gonadal sex differentiation) through to at least day 16.5. In sections of over-stained male gonads, *RALDH2 *expression was present in the developing seminiferous cords of the medulla (Fig. 6B). In females, *RALDH2 *expression was initially localised in the medulla of early embryos, but then declined in the medulla and became concentrated in the proliferating cortex at day 10.5, that is, at the onset of *STRA8 *up-regulation (Fig. 6B). This suggests a potential role in *STRA8 *induction. *RALDH2 *expression was maintained in the medulla of the right female gonad throughout development (no cortex forms). Real time RT-PCR confirmed robust *RALDH2 *mRNA expression in both sexes, but with higher expression males (data not shown).

**Figure 7:**
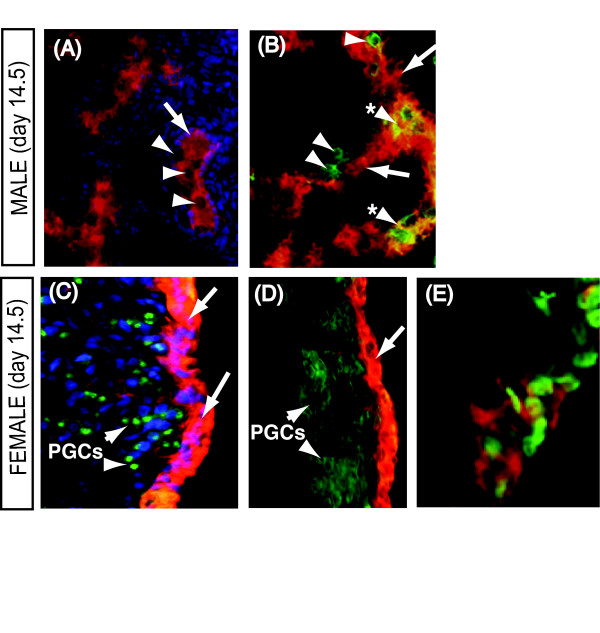
**Localisation of RALDH2 enzyme in day 14.5 male and female embryonic chicken gonads**. (A) In males, RALDH2 (arrows; red) localises in Sertoli cells of seminiferous cords. DAPI-stained nuclei delineate germ cells that appear negative for RALDH2 (arrowheads). (B) Double staining with the germ cell marker, GCNA, shows RALDH2 is somatic cells (Sertoli cells; red), but not in many germ cells (arrowheads, green). Some germ cells appear to stain for both proteins (yellow; asterisked arrowheads). (C) RALDH2 expression in somatic (pre-follicular) cells of the left cortex (arrows; red). SOX2+ germ cells (arrowheads; green) are negative for RALDH2. (D) Double-staining of the left ovarian cortex, showing an outer zone of RALDH2+ somatic cells (red; arrows) and underlying germ cells that are GCNA+ (PGCs; arrowheads; green). (E) Double staining of cortical somatic cells reveals GATA4+ nuclei (green) in cells that are also positive for RALDH2 in the cytoplasm (red).

To confirm the expression pattern of RALDH2, a chicken-specific antibody was used to localise the protein in day 14.5 gonads. Immunofluorescence confirmed RALDH2 expression in the Sertoli cells of seminiferous cords in the developing testis (Fig. 7). Most germ cells in seminiferous cords were RALDH2 negative, although double labelling with anti-GCNA and anti-RALDH2 suggested some co-localisation (Fig. 7). Similarly, in day 12.5 and 14.5 female gonads, RALDH2 protein was localised in the somatic (pre-follicular) cells of the ovarian cortex (Fig. 7C and 7D). In females, RALDH2 protein did not co-localise with either GCNA or SOX2, both germ cell markers (Fig. 7C and 7D). Double staining for the somatic marker, GATA4, and RALDH2 delineated somatic cells of the ovarian cortex with RALDH2+ cytoplasm and GATA4+ nuclei (Fig. 7E). These data indicate that the somatic cells (Sertoli cells of males and cortical pre-follicular cells of females) have the capacity to synthesise retinoic acid.

**Figure 8:**
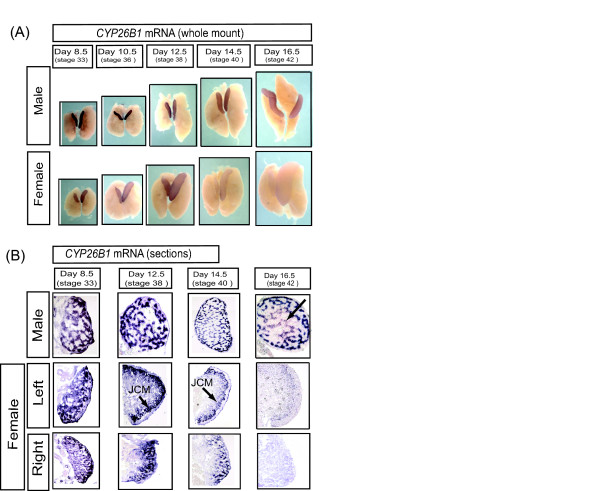
**Expression of *CYP26B1 *mRNA during gonadal development in chicken embryos, assessed by whole mount *in situ *hybridisation**. (A) In males, CYP26B1 is strongly expressed in both gonads, and not in mesonephric kidneys, throughout development. In females, expression is initially strong in both gonads, but declines from day 14.5. (B) In males, *CYP26B1 *expression localises to seminiferous cords in developing testes (arrow). In females, CYP26B1 is initially expressed in the medulla of both gonads, but becomes concentrated in the juxtacortical medulla (JCM) from day 12.5, and expression declines thereafter. The left cortex is always negative for CYP26B1 (e.g., arrow at 16.5). In the right gonad, expression persists in the medulla overdevelopment, declining at later stages.

**Figure 9:**
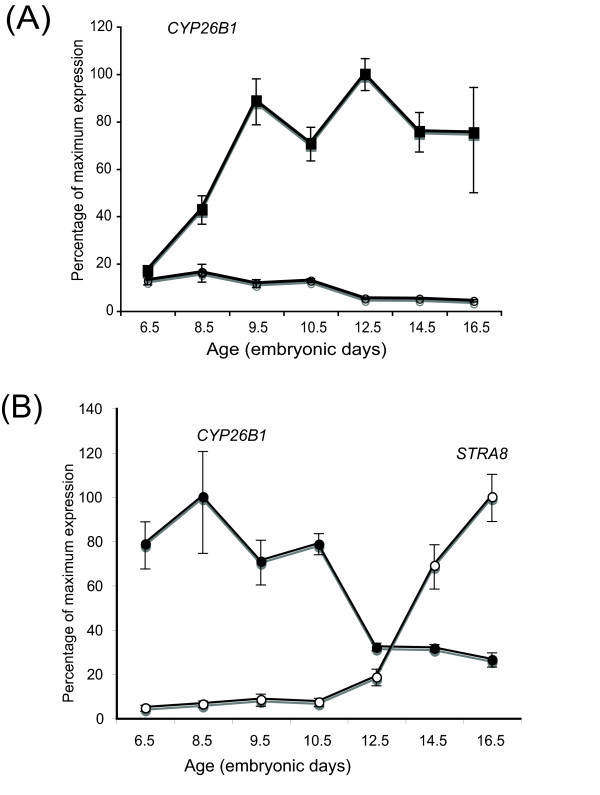
**Expression of *CYP26B1 *mRNA in embryonic chicken gonads, assessed by real time PCR (qRT-PCR)**. (A) *CYP26B1 *expression is up-regulated in male gonads from day 8.5 (closed squares). In female gonads (left and right pooled) there is initially a low level of expression, relative to males. From day 10.5, expression declines in females (open circles). (B) Gonadal expression profiles of *CYP26B1 *(closed circles) and *STRA8 *(open circles) in female chicken embryos, analysed together. An inverse relationship is evident; when *CYP26B1 *expression declines from day 10.5, *STRA8 *expression is up-regulated.

The key retinoic acid-degrading enzyme, *CYP26B1*, showed sexually dimorphic expression in embryonic gonads that correlated with the onset of meiotic prophase in females (Fig. 8). *CYP26B1 *mRNA was initially detectable in the gonads of both sexes at least as early as day 8.5 (stage 33), well prior to meiosis. Expression was maintained in males, but declined in females at the time of meiosis (Fig 8A). In sections of over-stained tissues, *CYP26B1 *mRNA was localised in the seminiferous cords, that is, in the same location as *RALDH2 *expression (Fig 8B). The pattern and extent of staining indicated expression at least in Sertoli cells. In female sections, *CYP26B1 *showed an intriguing profile. The gene was initially expressed in the gonadal medulla, as in males, but expression became progressively restricted to the juxtacortical medulla (JCM), adjacent to the cortex, and then declined (Fig. 8B). The developing cortex itself did not show *CYP26B1 *expression at any stage (Fig. 8B). Taken together with the *RALDH2 *data, these observations indicate that the developing ovarian cortex – the site of germ cell meiosis – has the capacity to synthesise retinoic acid, but does not express the retinoic acid-degrading enzyme, *CYP26B1*. In the right female gonad, *CYP26B1 *expression was localised to the medulla throughout development (Fig 8B), consistent with the absence of *STRA8 *expression in this location. Analysis of gonadal *CYP26B1 *by qRT-PCR suggested higher expression in males compared to females from early in development (from day 6.5) (Fig. 9A). This may reflect legitimate up-regulation in males as a result of male somatic commitment, or it may reflect the progressive restriction of expression in females, so that *CYP26B1*+ cells comprise a progressively smaller component of the growing ovary (see the restricted expression of *CYP26B1 *in later stage females is Fig 8B). When female *CYP26B1 *and *STRA8 *expression profiles are analysed together, an inverse relationship is apparent (Fig 9B). As *CYP26B1 *mRNA levels decline from day 10.5, *STRA8 *levels begin to increase, suggesting that an accumulation of retinoic acid may lead to *STRA8 *induction.

**Figure 10:**
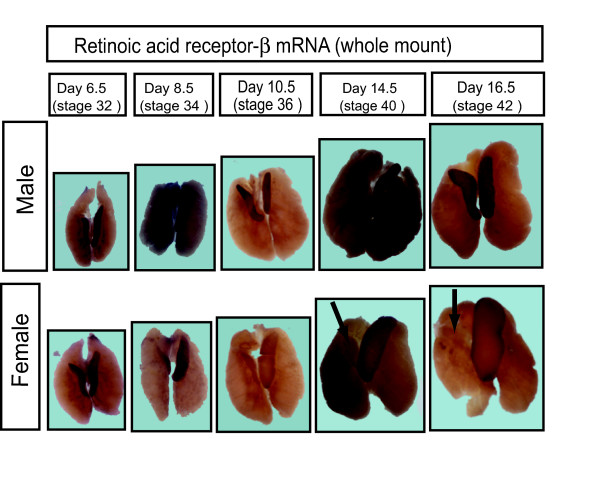
**Expression of Retinoic acid receptor β (RARβ) during gonadal development in chicken embryos, assessed by whole mount in situ hybridisation.** RARβ is expressed in both gonads of both sex throughout development, except in females, where expression declines in the right gonad (arrow).

Expression profiles of the other retinoic acid metabolising enzymes were also studied, using whole mount *in situ *hybridisation for chicken *RALDH1*, *RALDH3*, and *CYP26A1 *and *CYP26C1 *(Table 1). *RALDH1 *was expressed only in male gonads at all stages examined, from day 6.5 (stage 30) through to day 16.5 (stage 42). Similarly, *RALDH3 *was expressed male-specifically, but in a more temporally defined pattern, from E8.5 to E12.5. Only *RALDH2*, therefore, was expressed in the gonads of both sexes throughout development, including the time of meiosis induction in females. The other *CYP26 *mRNAs showed female biased expression in young gonads, but *CYP26A1 *and *CYP26C1 *were not expressed in the gonads of either sex from day 8.5 (Table 1). This excludes these enzymes as mediators of retinoic acid degradation during most of gonadal development. The only *CYP26 *mRNA showing a sexually dimorphic expression profile consistent with a role in meiosis was therefore *CYP26B1 *(Table 1). We also examined expression of the retinoic acid receptor β gene (Figure 10). Whole mount *in situ *hybridisation showed that RARβ was expressed in the gonads of both sexes throughout development. However, in the female, expression declined in the right gonad, from day 14.5 (Fig. 10; arrows).

**Table 1:** Dynamic expression of genes involved in retinoic acid synthesis and degradation in embryonic chicken gonads.

Retinoic acid degradation					Meiosis ⇒		
Gene	Sex	Day 6.5 (stage 31)	Day 8.5	Day 10.5	Day 12.5 (stage 38)	Day 14.5	Day 16.5
*RALDH1*	Female	-	-	-	-	-	-
	Male	-	++	+++	+++	+++	+++
*RALDH2*	Female	++	+++	+++	+++	+++	+++
	Male	+++	+++	+++	+++	+++	+++
*RALDH3*	Female	-	-	-	-	-	-
	Male	-	+++	+++	+++	-	-
							
Retinoic acid degradation					Meiosis ⇒		
Gene	Sex	Day 6.5 (stage 31)	Day 8.5	Day 10.5	Day 12.5 (stage 38)	Day 14.5	Day 16.5

*CYP26A1*	Female	+	-	-	-	-	-
	Male	-	-	-	-	-	-
*CYP26B1*	Female	+++	+++	+++	+++	+	-
	Male	+++	+++	+++	+++	+++	+++
*CYP26C1*	Female	+	-	-	-	-	-
	Male	-	-	+	+	-	-

**Expression is based on whole mount *in situ *hybridisation.** Overall level of gene expression was reflected in staining intensity; -not expressed, + lowly expressed; +++ strongly expressed. The onset of meiosis in females (SCP3 protein expression) is indicated by arrow.

## Discussion

This study provides the first molecular analysis of meiosis onset in an avian species, and presents evidence of a conserved role for retinoic acid in regulating entry into meiosis. *In vivo *histology and *in vitro *experiments conducted over thirty years ago indicated that female germ cells enter meiotic prophase during embryogenesis in birds, as occurs in mammals [24-26]. These early studies variably reported that meiosis in the left female chicken gonad commences on day 14, 15, 16, 17 or 18 of development [14,15,27]. We have found that chicken *STRA8 *is up-regulated specifically in the left female gonad from day 12.5, as assessed by qRT-PCR, and meiosis is detectable by SCP3 immunostaining in some cells at day 15.5, becoming widespread at day 16.5 and 17.5 (Fig. 2). This indicates that entry into meiosis is not a synchronous event in the chicken embryo, but occurs over a number of days. This has been reported previously, in a detailed study on the stages of meiosis in the germ cells of the left female gonad [14]. Therefore, while some cells may be entering meiotic prophase, others are still undergoing mitosis. Indeed, it has been reported that the germ cell population as a whole is still proliferating up to day 17 [25]. This implies that the signal for meiosis in the female chicken embryo is activated asynchronously in the germ cell population. In the mouse embryo, there is a anterior to posterior wave of meiosis in the female gonad over 24 – 48 hours [8,28]. However, this was not observed in the chicken (Fig's 2 and 3).

In contrast to the mouse, germ cell meiosis in the female chicken embryo occurs well after the onset of somatic gonadal sex differentiation, which begins at day 6 (stage 29) [22]. Ovarian differentiation is well advanced by the time that SCP3 protein is first detectable by immunofluorescence (day 15.5). Therefore, somatic commitment and meiosis are significantly separated in time. This is an interesting difference between chicken and mouse. Byskov [29] recognised mammals with so-called "immediate" versus "delayed" meiosis. In some species, meiosis occurs "immediately" at the time of somatic gonadal sex differentiation (such as the mouse), while meiosis is "delayed" in others, occurring after the onset of somatic differentiation (as in humans and rabbits). The chicken embryo would fall into the so-called "delayed" meiosis group. The reasons for this delay are unclear, although the germ cells could become committed to the female pathway at the molecular level at the time of gonadal sex differentiation in so-called "delayed" species, only showing morphological signs later in development. In the chicken and in other species, an aggregation of mitochondria occurs around the Golgi, called the Balbiani body, well prior to meiosis. In the chicken embryo, the Balbiani body is first detected in some female germ cells at day 7, and in many at day 10 [25], implying commitment to the female pathway shortly after the onset of gonadal sex differentiation (day 6). (A Balbiani body has recently been described in neonatal mouse oocytes; [30]).

In the female chicken embryo, it has been reported that pre-meiotic DNA synthesis begins in the germ cells of the left gonad between days 15 and 16 [31]. In the mouse, *Stra8 *is required for the pre-meiotic DNA synthesis that precedes meiosis [9]. Cloning and expression of chicken *STRA8 *revealed female-specific expression in the left ovarian cortex from day 12.5, prior to the onset of meiosis. This expression pattern suggests that, as in mouse, *STRA8 *is expressed in pre-meiotic germ cells and plays a role in meiotic induction in females. Notably, no *STRA8 *expression was detectable in the right female gonad, consistent with a lack of meiotic germ cells there. The organ culture studies described here indicate that germ cells in the left female gonad can enter meiosis normally at day 15.5 when explanted at day 10.5 and cultured in serum-free basal medium. This indicates that either the germ cells enter meiosis cell autonomously according to an intrinsic clock, or that a signal for meiosis is sent at or before the time of culture (day 10.5).

**Figure 11:**
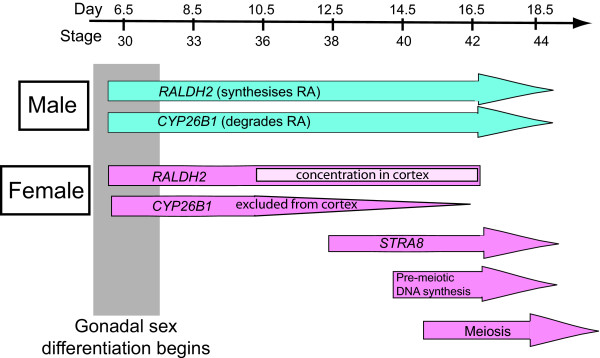
**Timing of meiosis and retinoic acid metabolism during gonadal development in chicken embryos**. Male shown in blue, female shown in pink. Gonadal sex differentiation begins at day 6.0. In the male gonads, *RALDH2 *and *CYP26B1 *are both highly expressed and meiosis does not occur during embryogenesis. In the left female gonad, *RALDH2 *is highly expressed, becoming concentrated in the cortex as it proliferates. *CYP26B1 *expression is down-regulated after E10.5, and is excluded form the cortex. At E12.5, STRA8 is up-regulated in the left gonad, and, at, E15, pre-meiotic DNA synthesis begins [31]. Meiosis is detectable histologically from E15.5.

It has been shown that retinoic acid triggers meiosis in female mouse embryos. In the study described here, we also find evidence of retinoic acid involvement in regulating avian meiosis. In the chicken embryo, RALDH2 and CYP26B1 are major enzymes of retinoic acid synthesis and degradation, respectively [23,32,33]. Embryonic chicken tissues that express *RALDH2 *have been shown to possess retinoic acid synthesising activity [32,34]. *RALDH2 *is therefore a robust indicator of retinoic acid synthesis in the chicken embryo, as in the mouse. Similarly, *CYP26B1 *expression reflects sites of retinoic acid catabolism [35]. Generally, *RALDH2 *and *CYP26B1 *are expressed in a co-ordinated fashion, usually in complimentary domains that result in localised patterns of retinoic acid action (where *RALDH2 *is expressed, *CYP26B1 *is not, and vice versa). The study reported here is the first detailed description of genes encoding retinoic acid metabolising enzymes in the embryonic gonads of an avian species. Furthermore, we find dynamic expression patterns that implicate retinoic acid signaling in avian meiosis, as in the mouse. Figure 11 summarises the expression of *RALDH2 *and *CYP26B1 *in relation to the initiation of meiosis in the chicken embryo. The two enzymes are strongly co-expressed in the developing testis, potentially leading to retinoic acid synthesis and degradation, with no accumulation of retinoic acid to influence germ cells. This is despite the strong expression of RAR-β throughout development in males. In the female, *RALDH2 *expression is maintained in the left gonad and *CYP26B1 *is down-regulated after day 10.5, pointing to an accumulation of retinoic acid prior to and at the time of *STRA8 *induction (Fig. 9B and Fig. 11). In the right female gonad, where only a medulla is present, both *RALDH2 *and *CYP26B1 *are expressed. This would lead to a degradation of retinoic acid, and indeed no meiosis is seen in the right gonad. Similarly, a small number of germ cells in the left female gonad remain scattered in the CYP26B1+ medulla (GCNA^+ ^cells seen in Fig. 2). These germ cells also do not enter meiosis (Fig 2). These expression patterns in the female support a role for retinoic acid in initiating meiosis.

However, *RALDH2 *expression was detectable in the left cortex of female gonads from early stages (at day 8.5 and day 10.5; figure 6B). Germ cells accumulate in the RALDH2+ left cortex as it proliferates at theses early stages, while *STRA8 *up-regulation is first detectable by qRT-PCR at day 12.5. The discrepancy in timing between cortical *RALDH2 *and *STRA8 *expression could indicate that retinoic acid acts indirectly to induce meiosis. Retinoic acid may set in motion a series of molecular events that result in *STRA8 *induction (In the mouse, it is assumed that retinoic acid directly activates *Stra8 *gene expression, but this has not been definitively shown). Embryonic chicken ovaries explanted in basal serum-free medium at day 10.5 could still initiate meiosis, implying that commitment to the female pathway had already occurred at the time of explantation. This commitment could be mediated by the early synthesis of retinoic acid in the developing cortex.

An alternative possibility is that is that retinoic acid synthesised by *RALDH2 *in the cortex is preferentially targeted to the underlying juxtacortical medulla, the site of *CYP26B1 *expression, bypassing the germ cells. In early stages (up to day 12.5), this would result in retinoic acid degradation, but, at later stages, loss of *CYP26B1 *expression would make retinoic acid available to the germ cells. Differential expression of retinoic acid receptors could be the mechanism for regulating the bioavailablity of retinoic acid to germ cells. We found that RAR-β is expressed in both sexes over development, with stronger expression males. The significance of RAR-β expression in males is unclear, but it is assumed that all synthesised retinoic acid is degraded. Despite the lower RAR-β expression in the left female gonad, differential RAR-β expression could mediate the timing of retinoic acid action. The precise sites of RAR expression within developing gonads were not examined here, but future studies should address this point.

**Figure 12:**
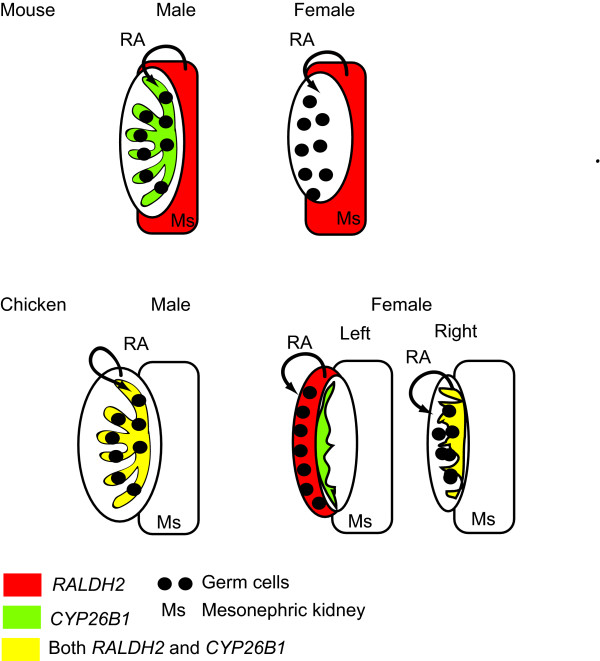
**A comparison of retinoic acid metabolism in the embryonic urogential system of the mouse and chicken.***RALDH2*-expressing tissue is shown in red, *CYP26B1*-expressing tissue is shown in green; tissues expressing both enzyme mRNAs are shown in yellow. In the mouse at E12.5, *Raldh2 *is expressed in the mesonephric kidney, retinoic acid then enters the gonad and in males, is degraded by Cyp26b1. In females, Cyp26b1 is absent, allowing retinoic acid to induce germ cells meiosis. In the chicken at E14.5, the gonads of both sexes express *RALDH2*. In males, *RALDH2 *and *CYP26B1 *are co-expressed in seminiferous cords. In the cortex of the left female gonad, where germ cells are located, *RALDH2 *is expressed but *CYP26B1 *is not (*CYP26B1 *expression is localised at the border between the cortex and medulla). In the right gonad, as in the testis, both *RALDH2 *and *CYP26B1 *are expressed, potentially leading to retinoic acid degradation and no meiosis.

Figure 12 compares gene expression in chicken and mouse embryonic gonads in relation to meiosis. Unlike the mouse embryo, in which *Raldh2 *is expressed in the adjoining mesonephric kidneys, chicken *RALDH2 *is expressed predominantly in the gonads themselves. In the male gonad, both *RALDH2 *and *CYP26B1 *are expressed in the developing seminiferous cords at all stages examined, from day 6.5 to day 16.5. Immunofluorescence confirmed Sertoli cell expression for RALDH2. Therefore, the seminiferous cords are expected to both synthesise and degrade retinoic acid, preventing it from influencing germ cells (Fig 12). Retinoic acid has been shown to be a potent stimulator of *CYP26B1 *gene expression in the chicken embryo [35], which would provide a mechanism of negative auto-regulation in the seminiferous cords. *RALDH2 *is also expressed throughout development in female gonads, initially in the medulla of both left and right gonads, but becoming concentrated in the proliferating cortex of the left gonad. This is the site of meiosis. In contrast, in the left female gonad, *CYP26B1 *expression becomes localised to the juxtacortical medulla – but never in the cortex. Therefore, in the left female gonad, retinoic acid synthesised but not degraded in the cortex could regulate entry of cortical germ cells into meiosis, either directly or indirectly (Fig. 12). In the mouse, *Cyp26b1 *is extinguished completely in female gonads at the time of meiosis.

In the female chicken embryo, the fate of germ cells is dictated by their location; those in the cortex of the left gonad enter meiosis, while those in the right gonad and those in the left medulla fail to enter meiosis. This indicates that a somatic signal, rather than cell autonomous mechanism, regulates entry into meiosis in the chicken embryo. The asymmetry of germ cell fate in the female chicken embryo therefore provides a unique system for testing the regulation of meiosis. If retinoic acid plays a role in avian meiosis, some aspect of retinoic acid metabolism is expected to show asymmetry between the left and right gonads. This asymmetry is evidently achieved by proliferation of a thickened ovarian cortex in the left gonad, which strongly expresses *RALDH2 *but not *CYP26B1*. In the left gonad, germ cells become concentrated in this cortical environment. In contrast, germ cells of the right gonad and the left medulla are "stranded" in an environment expressing *CYP26B1*, potentially leading to degradation of retinoic acid (Fig. 12). Germ cells would therefore not enter meiosis. Indeed, it has been shown that germ cells in the right gonad of female chicken embryos undergo cell death [36]. In males, an unidentified signal from the somatic cells is presumed to induce mitotic arrest, as occurs in mouse. This signal is presumably absent in the both the right and left female chicken gonads.

The data presented here indicate the left ovarian cortex is critical for meiosis in female chicken embryos. This is supported by previous organ culture experiments. Erickson [25] cultured whole female embryonic gonads, isolated pieces of cortex, or groups of pure germ cells. Germ cells entered meiosis when present in the intact gonads, in isolated cortex, and when isolated and co-cultured with pieces of cortex, but not when isolated and cultured alone. It was concluded that "the differentiation of female germ cells is regulated by the somatic cells of the cortex" [25]. The data presented here support this conclusion and suggest that retinoic acid is the factor associated with the somatic cells of the cortex. The organ culture experiments conducted here indicate that germ cells commit to the female pathway at or prior to day 10.5 (the time of explantation in serum-free basal medium). This is consistent with the proliferation of a *RALDH2*+ cortex at this time (Fig. 11).

An alternative possibility is that germ cells enter meiosis cell-autonomously in birds. In a previous study, primordial germ cells were isolated from quail blastoderms and introduced into very young (day 3) chicken embryos *in ovo*. These quail germ cells populated the chicken embryonic gonads and entered meiosis according to the quail timetable (meiotic figures from day 12.5) rather than the chicken timetable (meiotic figures from day 15.5) [37]. This suggested that meiosis follows an endogenous clock in avian (quail) germ cells. However, as mentioned above, the left-right asymmetry of germ cell fate in the female chicken embryo argues for differential somatic signals rather than a cell autonomous mechanism. An alternative interpretation of the quail transplantation experiment is that retinoic acid from the chicken gonad is able to induce meiosis in the quail cells because they are responsive to it at an earlier time than the endogenous chicken germ cells.

This study presents the first detailed analysis of meiosis onset in avian embryos. We provide circumstantial evidence that retinoic acid has a conserved role in regulating entry into meiosis, at least in higher vertebrates. Further studies are now required to test this possible role for retinoic acid in avian meiosis. Such studies should include an examination of meiosis in isolated germ cells or in whole gonads cultured in the presence of retinoic acid or its inhibitors.

## Abbreviations

BSA: Bovine serum albumen; CHAPS: 3-[(3-Cholamidopropyl) dimethyl-ammonio]-1-propane sulfonate; C*YP26*: Cytochrome p450 from family 26; FCS: foetal calf serum; GCNA: germ cell nuclear antigen; NTMT: sodium chloride: Tris base: magnesium chloride with Tween 20; *RALDH*: Retinaldehyde dehydrogenase; qRT-PCR: quantitative reverse transcription and polymerase chain reaction; RT-PCR: Reverse transcription and polymerase chain reaction; SCP3: Synaptonemal complex 3; *STRA8*: Stimulated by Retinoic Acid gene 8.

## Authors' contributions

CAS and JB designed the study, while CAS and KNR carried out the experiments (CAS performed the immunofluorescence, the organ culture, and some of the whole mount *in situ *hybridisation experiments. KNL carried out whole mount *in situ *hybridisation and quantitative real time PCR experiments). CAS, KNR, JB, PK and AHS prepared the manuscript. All authors read and agreed with the final draft.
